# Supplementary material for: Causal associations between cardiorespiratory fitness and type 2 diabetes
Source: Nat Commun. 2023 Jul 3;14:3904. doi: 10.1038/s41467-023-38234-w (PMC10318084; doi:10.1038/s41467-023-38234-w)
Supplement: Supplementary file 1 — Supplementary Information [file 41467_2023_38234_MOESM1_ESM.pdf]

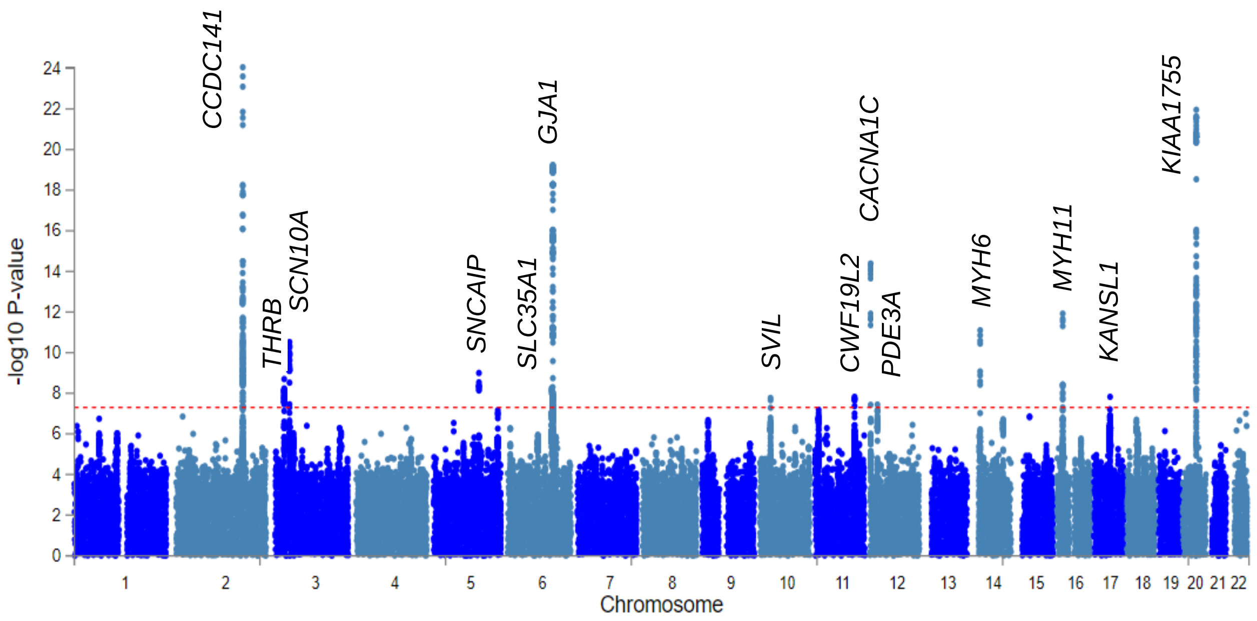


**(a)**

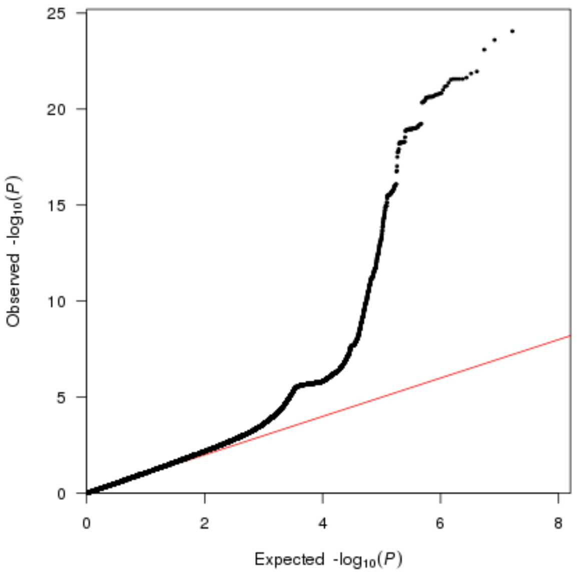


**(b)**

**Supplementary Figure 1. Manhattan plot (a) and Q-Q plot (b) of genome-wide association analyses on cardiorespiratory fitness among up to 69,416 participants in UKBB.** (a) 14 independent genome-wide significant loci are annotated. On the Manhattan plot, red horizontal line indicates genome-wide significance threshold (p < 5 x 10-8); (b) On the Q-Q plot, the red diagonal line indicates null association estimate. LD score regression estimated $\lambda_{GC}$=1.15, LD score regression intercept = 1.02 (95% CI 1.00 -1.04), suggesting that apparent inflation evident in the QQ-plot can be attributed to polygenicity of cardiorespiratory fitness.

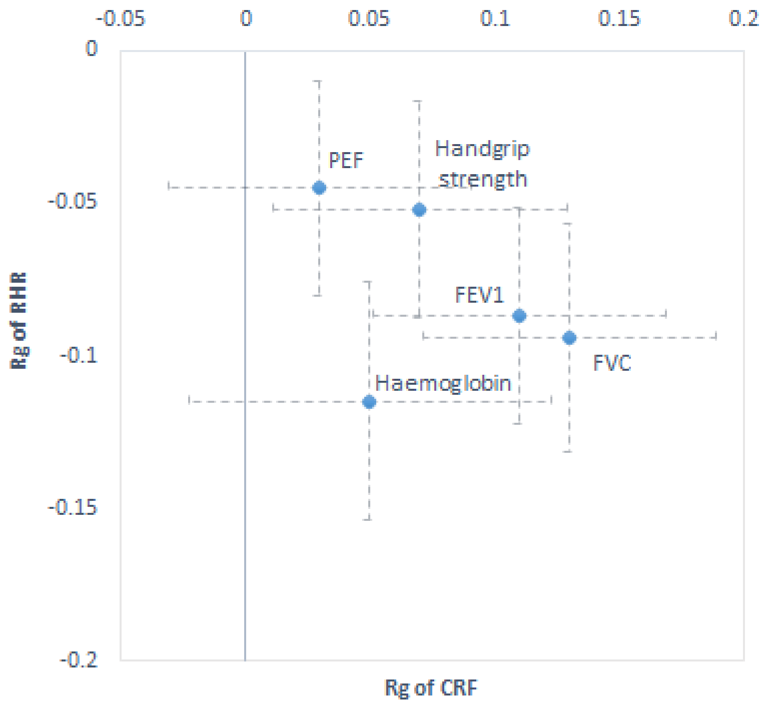


**Supplementary Figure 2. Comparison of genetic correlations between cardiorespiratory fitness (CRF), resting heart rate (RHR) and various physiologically relevant traits, including forced expiratory volume 1 (FEV1), forced vital capacity (FVC), peak expiratory flow (PEF), handgrip strength, and haemoglobin.**

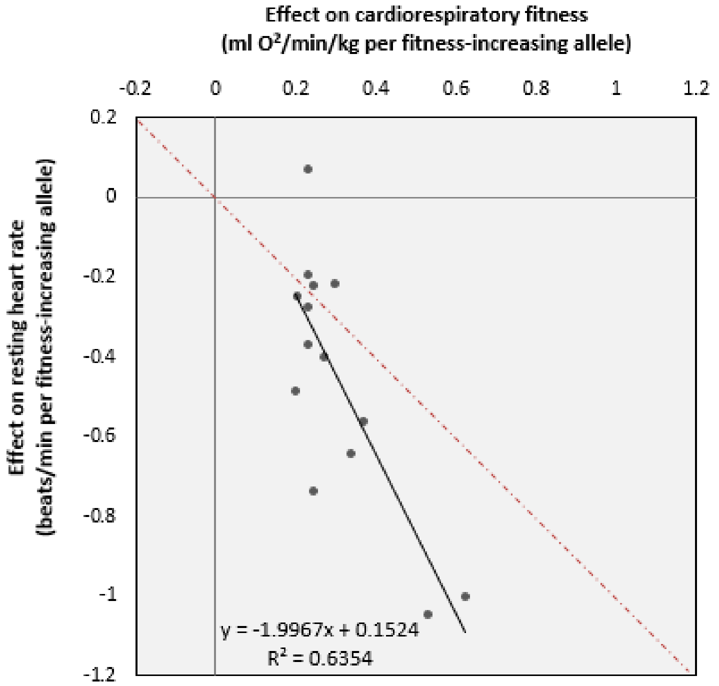


**(a)**

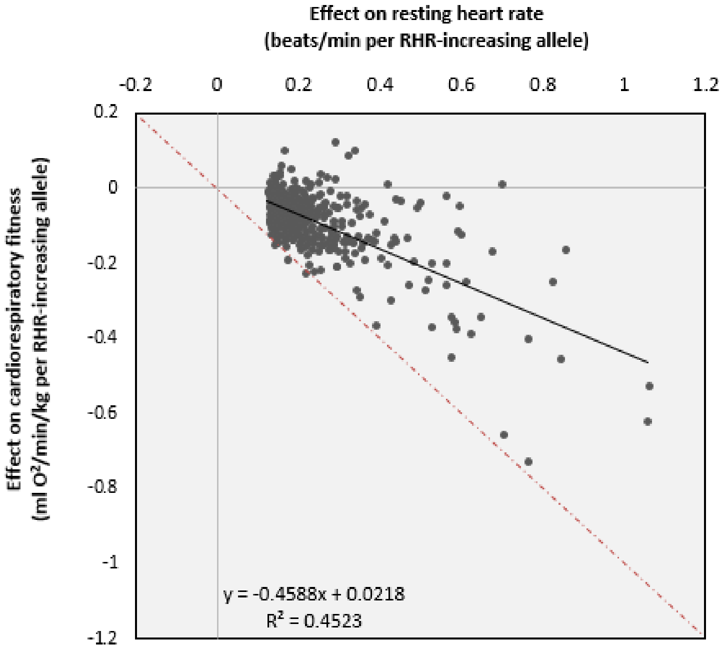


**(b)**

**Supplementary Figure 3. Comparison of effect of cardiorespiratory fitness (CRF) and resting heart rate (RHR)-associated independent variants ($p < 5 \times 10^{-8}$) in UK Biobank.** (a) effect of 14 distinct genome-wide significant CRF associated variants (effect alleles aligned to CRF-increasing) on RHR in UK Biobank (n=69,416); (b) effect of 427 distinct genome-wide significant RHR associated variants (effect alleles aligned to RHR-increasing) on CRF in UK Biobank (n= 452,941).

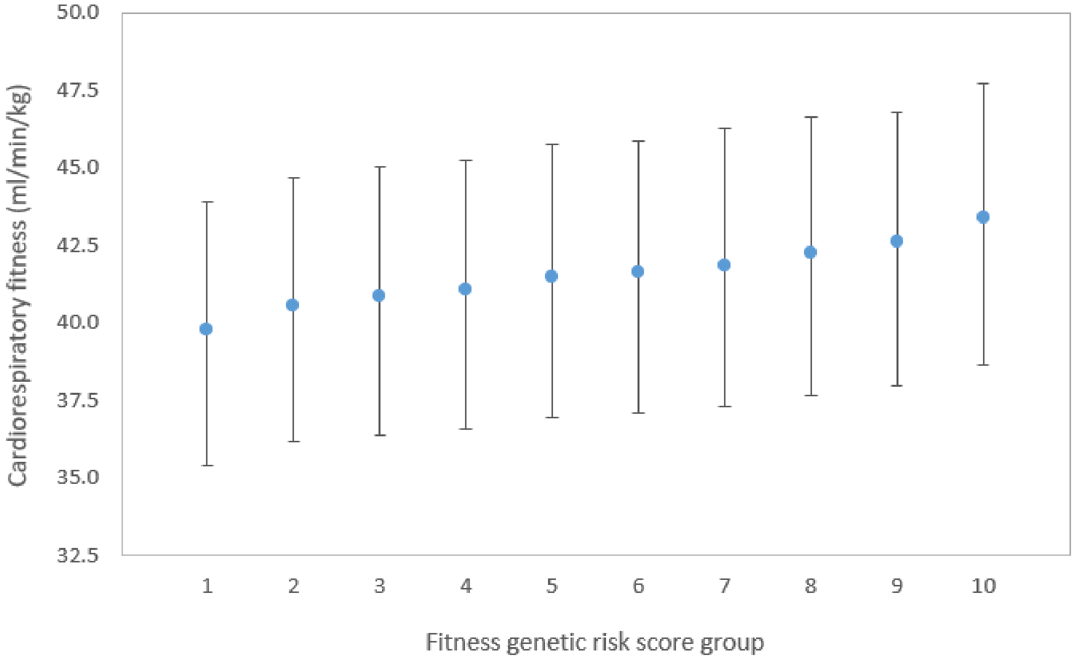


**Supplementary Figure 4. Mean (and 95% confidence interval) cardiorespiratory fitness levels by decile groups of genetic risk score constructed using 160 independent genome-wide significant variants in the UK Biobank study (n=69,416).** The mean difference between the highest GRS group and the lowest is 3.6 ml $O_2$ $min^{-1}$ $kg^{-1}$

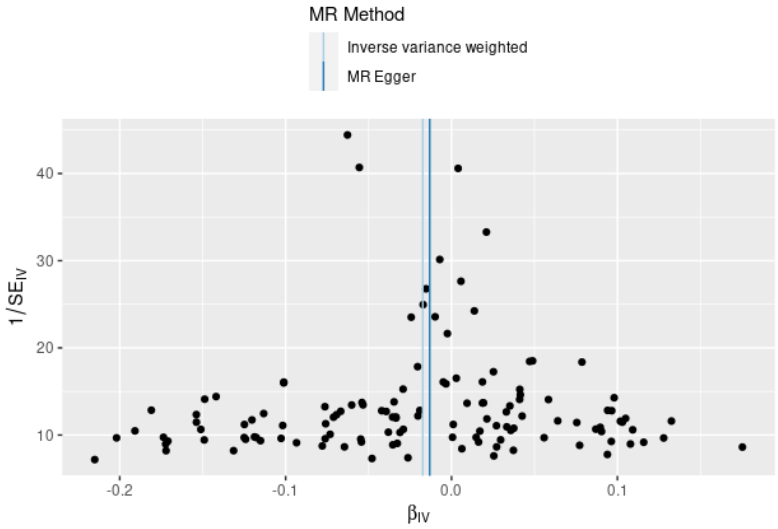


(a)

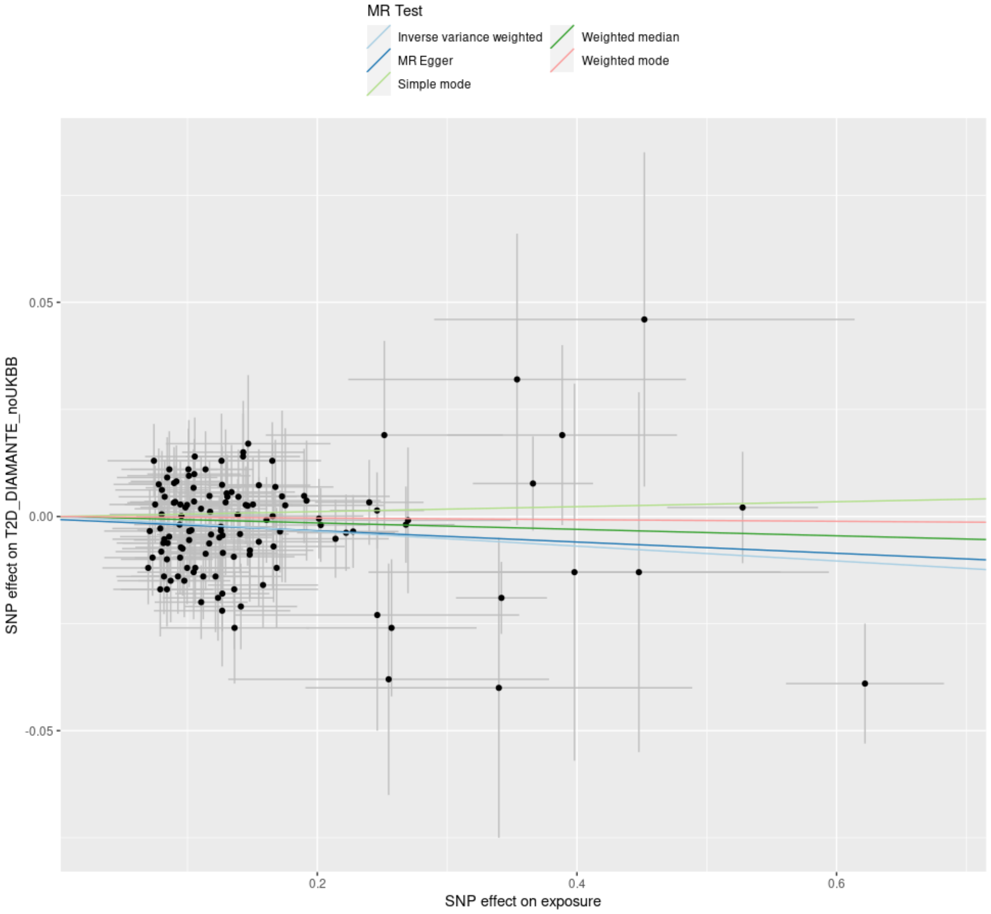


(b)

**Supplementary Figure 5 (a) Funnel plot for MR analyses of Radial filtered fitness score on T2D. (b) Scatter plot for various MR analyses of Radial filtered fitness score on T2D with slope indicates effect estimate of causal associations.** After removing outliers using Radial method, $I^2$ =0.0%, p-value for Cochran's Q = 0.686.

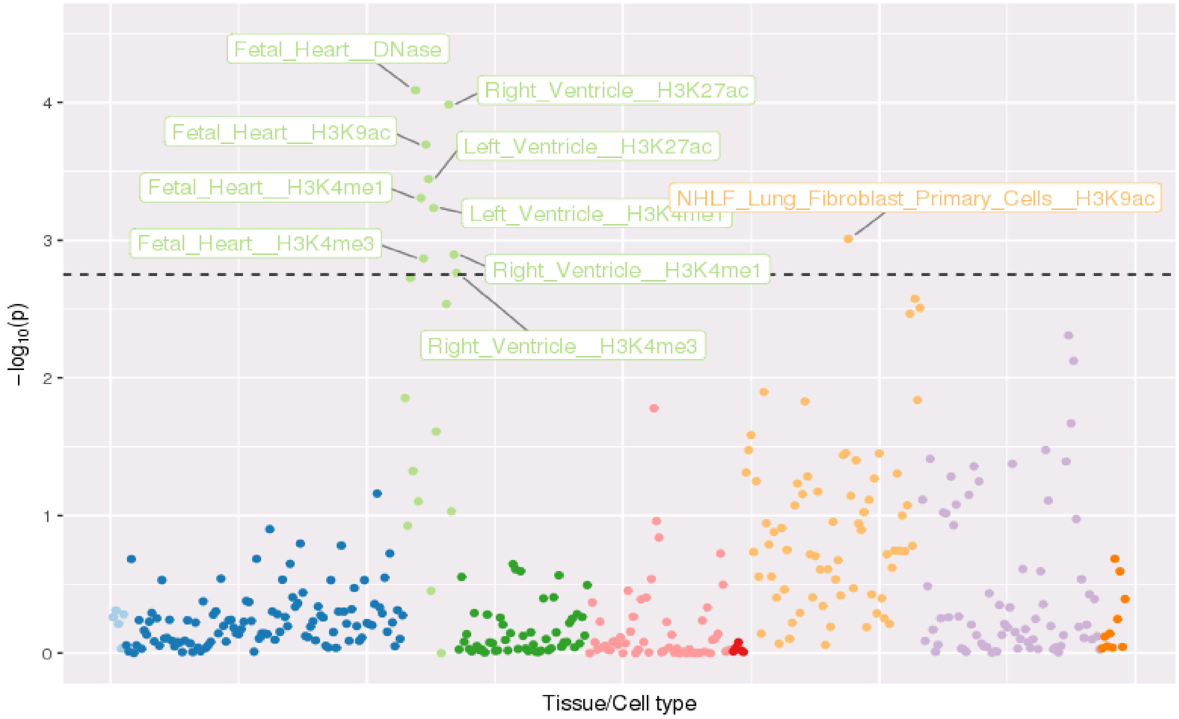


**Supplementary Figure 6. Tissue-specific enrichment for cardiorespiratory fitness GWAS using LD score regression applied to specifically expressed genes (LDSC-SEG).** Each dot represents a tissue or cell type further categorised into 9 general tissue groups indicated by different colours. Expression enrichment in 396 ROADMAP Epigenetics chromatin data. Horizontal dash line represents the significance threshold of false discovery rate = 0.05, –log10 (P) = 2.85 (chromatin). Any tissue and cell type above the line is considered enriched.

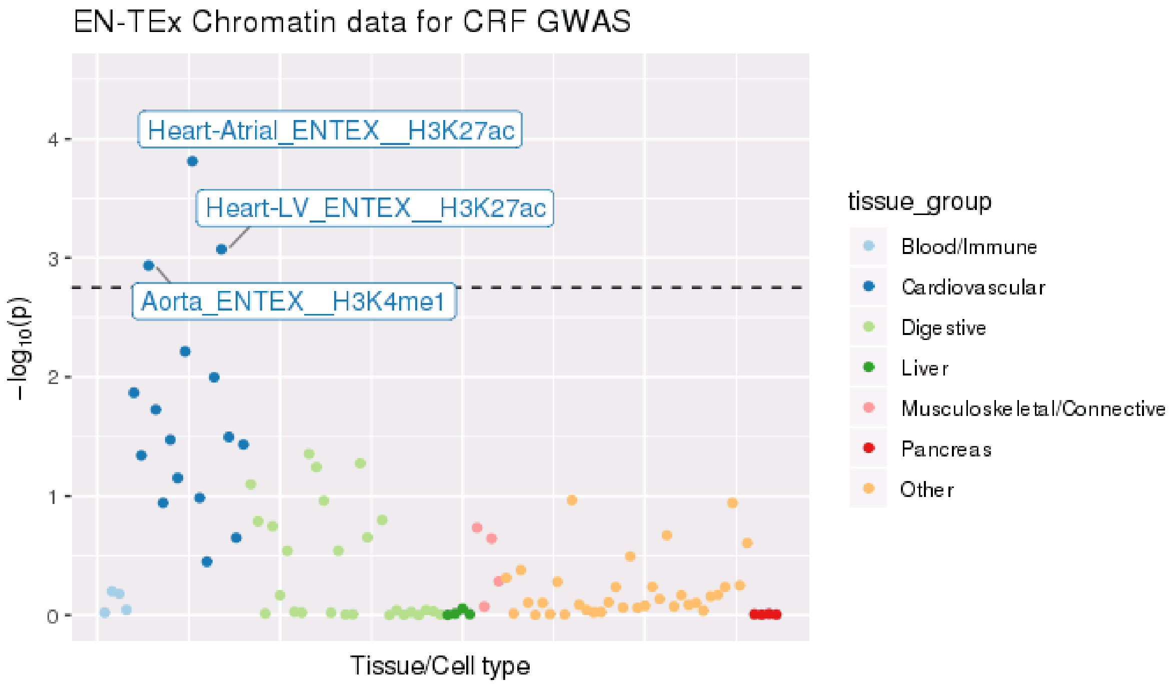


**Supplementary Figure 7. LD score regression applied to specifically expressed genes (LDSC-SEG) EN-TEx chromatin enrichment results for cardiorespiratory fitness GWAS.** A total of 93 labels of tissue and cell types are included in the analyses. Each dot represents a tissue or cell type categorised into 8 tissue groups marked in different colours. Horizontal dash line represents the significance threshold of false discovery rate = 0.05, (a) –log10(P) = 2.75 (gene expression). (b) –log10 (P) = 2.85 (chromatin). Any tissue and cell type above the line is considered enriched.

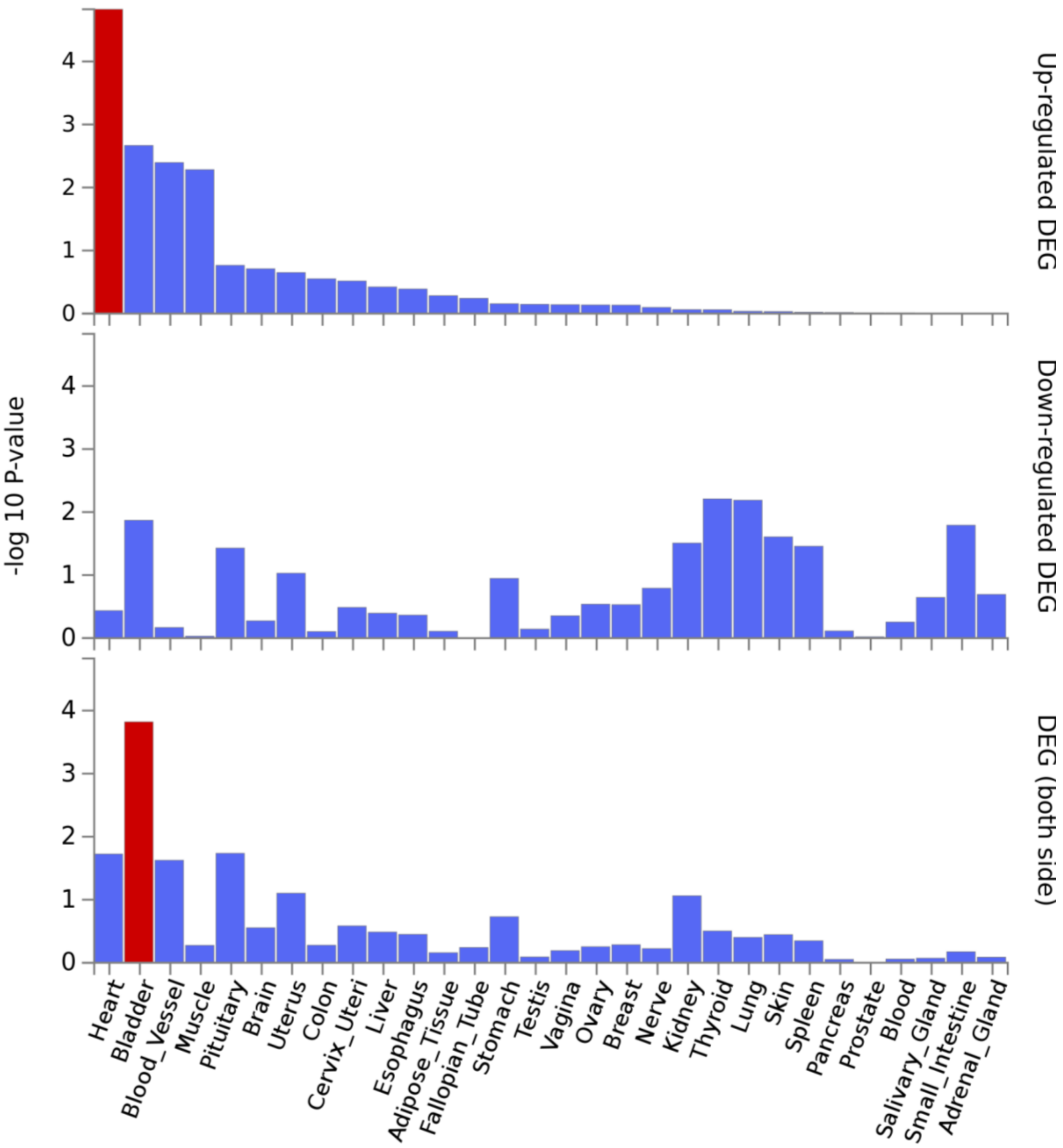


**Supplementary Figure 8. Tissues enriched with differentially expressed genes sets identified using prioritised genes associated with cardiorespiratory fitness.** Output from FUMA using GTEx v8 30 general tissue types.

**Supplementary Table 1. UK Biobank participant characteristics of those with cardiorespiratory fitness estimates from exercise testing, stratified by sex and incident type 2 diabetes status.** Participants with prevalent type 2 diabetes at baseline were excluded from this analysis.

| **Sex** | **Women** | | | **Men** | | |
|---|---|---|---|---|---|---|
| **T2D status** | Pooled | Non-T2D | T2D | Pooled | Non-T2D | T2D |
| **N** | 39,395 | 38,663 | 732 | 34,179 | 33,059 | 1,120 |
| **Age (y)** | 57 ± 8 | 57 ± 8 | 60 ± 8 | 58 ± 8 | 58 ± 8 | 60 ± 8 |
| **Height (cm)** | 163 ± 6 | 163 ± 6 | 161 ± 6 | 176 ± 7 | 176 ± 7 | 174 ± 7 |
| **Weight (kg)** | 70.1 ± 13.0 | 69.9 ± 12.9 | 80.7 ± 16.7 | 84.9 ± 13.3 | 84.6 ± 13.1 | 92.6 ± 15.9 |
| **BMI (kg·m$^2$)** | 26.4 ± 4.7 | 26.4 ± 4.6 | 30.9 ± 5.8 | 27.4 ± 3.8 | 27.3 ± 3.8 | 30.4 ± 4.7 |
| **FFM (kg)** | 44.1 ± 4.8 | 44.1 ± 4.7 | 46.6 ± 6.2 | 63.2 ± 7.5 | 63.1 ± 7.4 | 65.3 ± 8.5 |
| **Waist circumference (cm)** | 83.1 ± 11.6 | 82.9 ± 11.5 | 94.5 ± 12.8 | 95.5 ± 10.6 | 95.2 ± 10.4 | 103.7 ± 12.1 |
| **Resting heart rate (bpm)** | 67 ± 10 | 67 ± 10 | 70 ± 11 | 65 ± 11 | 65 ± 11 | 68 ± 12 |
| **Systolic blood pressure (mmHg)** | 130 ± 17 | 130 ± 17 | 137 ± 17 | 135 ± 16 | 135 ± 16 | 139 ± 16 |
| **Diastolic blood pressure (mmHg)** | 78 ± 10 | 78 ± 10 | 81 ± 10 | 81 ± 9 | 81 ± 9 | 82 ± 10 |
| **$VO_2$max (ml $O_2$ min$^{-1}$ kg$^{-1}$ FFM)** | **39.8 ± 7.1** | **39.8 ± 7.0** | **36.6 ± 6.8** | **43.1 ± 6.4** | **43.2 ± 6.3** | **40.0 ± 5.9** |
| **$VO_2$max (ml $O_2$ min$^{-1}$ kg$^{-1}$ body mass)** | 25.5 ± 5.7 | 25.6 ± 5.7 | 21.6 ± 4.8 | 32.4 ± 5.8 | 32.5 ± 5.8 | 28.6 ± 5.1 |

Values are means ± standard deviations. T2D: Type 2 diabetes. BMI: Body mass index. FFM: Fat-free mass. $VO_2$max: Maximal oxygen consumption.

**Supplementary Table 2. Sequentially adjusted logistic regression models of the association of sex- and age-residualised cardiorespiratory fitness with incident type 2 diabetes in UK Biobank.** Participants with prevalent type 2 diabetes at baseline were excluded from analyses.

| | Per ml $O_2 \cdot min^{-1} \cdot kg^{-1}$ fat-free mass | | |
|---|---|---|---|
| | **Pooled** | **Women** | **Men** |
| **Model 1** | 0.94 (0.93 to 0.94) [$4x10^{-4}$] | 0.95 (0.94 to 0.96) [$9x10^{-3}$] | 0.93 (0.92 to 0.94) [$5x10^{-3}$] |
| **Model 2** | 0.96 (0.95 to 0.97) 5[$x10^{-13}$] | 0.97 (0.96 to 0.98) [$3x10^{-7}$] | 0.95 (0.94 to 0.96) [$1x10^{-10}$] |
| **Model 3** | 0.97 (0.96 to 0.98) [$5x10^{-9}$] | 0.97 (0.96 to 0.98) [$1x10^{-5}$] | 0.97 (0.96 to 0.98) [$8x10^{-6}$] |
| | | | |
| | **Per ml $O_2 \cdot min^{-1} \cdot kg^{-1}$ body mass** | | |
| | **Pooled** | **Women** | **Men** |
| **Model 1** | 0.88 (0.87 to 0.89) [$1x10^{-4}$] | 0.88 (0.87 to 0.89) [$3x10^{-3}$] | 0.88 (0.87 to 0.89) [$2x10^{-3}$] |
| **Model 2** | 0.91 (0.90 to 0.92) [$7x10^{-20}$] | 0.91 (0.90 to 0.92) [$7x10^{-14}$] | 0.91 (0.90 to 0.92) [$4x10^{-16}$] |
| **Model 3** | 0.96 (0.95 to 0.97) [$9x10^{-8}$] | 0.96 (0.94 to 0.98) [$7x10^{-5}$] | 0.96 (0.95 to 0.98) [$7x10^{-5}$] |

Values are odds ratios (95% confidence intervals). Figures in square brackets are exact p values. CRF: Cardiorespiratory fitness. COPD: Chronic obstructive pulmonary disease.

Model 1 is adjusted for age and sex.

Model 2 is additionally adjusted for ethnicity (White, other), hypertension (yes/no), history of stroke (yes/no), history of heart failure (yes/no), history of heart disease (yes/no), history of atrial fibrillation (yes/no), history of COPD (yes/no), history of cancer (yes/no), betablocker use (yes/no), calcium channel blocker use (yes/no), ACE inhibitor use (yes/no), Diuretic use (use (yes/no), bronchodilator use (yes/no), lipid-lowering agent (yes/no), iron deficiency medication (yes/no), smoking (never, former, current), alcohol consumption (never, former, current), meat intake, oily fish intake, fruit and vegetable intake, salt intake, employment (yes/no), and Area Deprivation Index.

Model 3 is additionally adjusted for adiposity (fat mass for fat-free mass scaled fitness, BMI for body mass scaled fitness).

**Supplementary Table 3. Results for the 14 independent genome-wide significant loci associated with cardiorespiratory fitness in European ancestry individuals in the UK Biobank study (N = 69,416).**

| Lead SNP | chr | pos | EA | OA | EAF | beta (s.e.) | p | Function | Nearest Gene* |
|---|---|---|---|---|---|---|---|---|---|
| **rs142556838** | 2 | 179747068 | C | T | 0.91 | 0.62 (0.06) | **1.6E-24** | intron | CCDC141 |
| **rs4811602** | 20 | 36849088 | G | A | 0.53 | -0.34 (0.04) | **1.7E-22** | intron | KIAA1755 |
| **rs1012020** | 6 | 122110346 | A | G | 0.90 | 0.52 (0.06) | **1.1E-19** | intergenic | GJA1 |
| **rs11062107** | 12 | 2187041 | A | G | 0.83 | -0.37 (0.05) | **2.5E-15** | intron | CACNA1C |
| **rs3915425** | 16 | 15912544 | T | C | 0.68 | 0.27 (0.04) | **8.2E-13** | intron | MYH11 |
| **rs422068** | 14 | 23864804 | T | C | 0.64 | 0.25 (0.04) | **1.1E-11** | intron | MYH6 |
| **rs6599251** | 3 | 38785809 | G | T | 0.45 | 0.23 (0.03) | **7.4E-11** | intron | SCN10A |
| **rs111299422** | 5 | 121868475 | D | I | 0.70 | -0.23 (0.04) | **1.4E-09** | intergenic | SNCAIP |
| **rs1561076** | 3 | 24497993 | A | G | 0.68 | -0.23 (0.04) | **2.0E-09** | intron | THRB |
| **rs4754197** | 11 | 107096785 | G | A | 0.78 | -0.24 (0.04) | **9.5E-09** | intergenic | CWF19L2 |
| **rs17695155** | 10 | 29820758 | T | C | 0.60 | 0.20 (0.04) | **1.1E-08** | intron | SVIL |
| **rs1572226** | 6 | 118635079 | C | G | 0.49 | -0.20 (0.03) | **1.4E-08** | intron | SLC35F1 |
| **rs62070949** | 17 | 44308053 | A | G | 0.84 | -0.30 (0.05) | **1.7E-08** | intergenic | KANSL1 |
| **rs67622553** | 12 | 20454434 | G | T | 0.77 | -0.23 (0.04) | **4.7E-08** | intergenic | PDE3A |

* Nearest genes for the intergenic variants were selected as the genes located closest to the variants that were also mapped by either eQTL or CI by FUMA. Chr: chromosome; pos: position; EA: effect allele; OA: other allele; EAF: effect allele frequency

**Supplementary Table 4. Genetic correlation between cardiorespiratory fitness, resting heart rate and several physiologically relevant traits using LD score regression.**

| Traits | CRF (UK Biobank) | | | RHR (UK Biobank) | | |
|---|---|---|---|---|---|---|
| | Rg | s.e. | p-value | Rg | s.e. | p-value |
| **CRF (Fenland)** | 0.78 | 0.17 | 3.1E-06 | -0.43 | 0.12 | 4.2E-04 |
| **RHR (UK Biobank)** | -0.68 | 0.03 | 5.4E-120 | -- | -- | -- |
| **Handgrip strength** | 0.07 | 0.03 | 0.016 | -0.01 | 0.02 | 0.442 |
| **FEV1** | 0.11 | 0.03 | 3.7E-04 | -0.09 | 0.02 | 2.5E-06 |
| **FVC** | 0.13 | 0.03 | 1.9E-05 | -0.09 | 0.02 | 6.8E-07 |
| **FEV1/FVC** | -0.03 | 0.03 | 0.290 | 0.004 | 0.02 | 0.814 |
| **PEF** | 0.03 | 0.03 | 0.420 | -0.05 | 0.02 | 0.012 |
| **Haemoglobin** | 0.05 | 0.04 | 0.148 | -0.12 | 0.02 | 3.7E-09 |

CRF: cardiorespiratory fitness; RHR: resting heart rate; FEV1: forced expiratory volume1; FVC: forced vital capacity; PEF: peak expiratory flow rate

**Supplementary Table 5. Validity of the four constructed genetic instruments for cardiorespiratory fitness in the Fenland study.**

| Instrument* | n_snp† | Association with measured fitness in Fenland | | | | | 2-sample MR‡ | | | |
|---|---|---|---|---|---|---|---|---|---|---|
| | | beta | s.e. | t | p | $AdjR^2$ | beta | s.e. | p | CochQp |
| 1 | 14 | 1.42 | 0.19 | 7.36 | 2.0E-13 | 0.54% | 1.40 | 0.19 | 5.7E-06 | 0.06 |
| 2 | 370 | 1.08 | 0.13 | 8.36 | <2.6E-16 | 0.70% | 1.09 | 0.13 | 6.5E-16 | 0.05 |
| 3 | 148 | 1.04 | 0.14 | 7.23 | 5.4E-13 | 0.52% | 1.04 | 0.14 | 2.1E-11 | 0.02 |
| 4 | 160 | 1.23 | 0.12 | 10.40 | <2.6E-16 | 1.08% | 1.15 | 0.11 | 7.6E-19 | 0.01 |

* All instrument effects were aligned as fitness-increasing.

*Instrument 1*: 14 independent, genome-wide significant variants associated with exercise-based fitness in UK Biobank ($p < 5 \times 10^{-8}$);

*Instrument 2*: 370 genome-wide significant RHR-associated variants that passed the Radial test and available in the Fenland genotyping data;

*Instrument 3*: 148 Radial-filtered RHR-associated variants that were also nominally significant in exercise-based fitness GWAS results in UK Biobank ($p < 0.05$);

*Instrument 4*: the combined fitness score: 160 independent variants combining the list of variants from (1) and (3); variant pairs in LD ($r^2 > 0.01$) were identified and the variants selected as independent top fitness variants (among (1)) were preferred.

† Only SNPs available in the Fenland study were included in the analyses, which is the reason why the number of SNPs listed here do not match the number of SNPs included in the instruments constructed in the UK Biobank.

‡ 2-sample MR approach: the genetic instruments of fitness in UK Biobank were treated as the exposure, fitness GWAS in the Fenland study was the outcome.

**Supplementary table 6. Results of Mendelian randomisation analyses between cardiorespiratory fitness and type 2 diabetes.**

| exposure* | nsnp | method | beta | s.e. | p | CochQp |
|---|---|---|---|---|---|---|
| Combined fitness score | 157 | Inverse variance weighted | -0.028 | 0.016 | 0.086 | <0.001 |
| | 157 | MR Egger | 0.003 | 0.036 | 0.937 | |
| | 157 | Egger Intercept | -0.005 | 0.005 | 0.330 | |
| | 157 | Weighted median | -0.007 | 0.010 | 0.442 | |
| | 157 | Penalised weighted median | -0.008 | 0.009 | 0.400 | |
| | 157 | MR-PRESSO Raw | -0.029 | 0.017 | 0.085 | |
| | 148 | MR-PRESSO Outlier-corrected | -0.019 | 0.008 | **0.016** | |
| | 160 | One-sample MR† | -0.037 | 0.007 | **8.8E-08** | |
| Radial filtered fitness score | 126 | Inverse variance weighted | -0.017 | 0.006 | **0.005** | 0.686 |
| | 126 | MR Egger | -0.014 | 0.013 | 0.274 | |
| | 126 | Egger Intercept | -0.001 | 0.002 | 0.780 | |
| | 126 | Weighted median | -0.009 | 0.010 | 0.373 | |
| | 126 | Penalised weighted median | -0.008 | 0.010 | 0.412 | |
| | 126 | One-sample MR† | -0.022 | 0.008 | **0.005** | |

GWAS summary statistics of the outcome type 2 diabetes were obtained from Mahajan *et al*.[96] with UK Biobank cohort depleted

Effect sizes are log Odds of diabetes per unit higher fitness (ml O2·$min^{-1}$·$kg^{-1}$ FFM)

* The combined fitness score used here contains 157 variants instead of 160 because only 121 variants were directly available in the type 2 diabetes GWAS, 36 proxies (LD > 0.8) were found using UK Biobank Random 25K unrelated White British cohort as the reference panel; The Radial filtered fitness score was obtained by conducting Radial-filtering from fitness to type 2 diabetes first, removing 34 variants as outliers, hence, 126 variants remained for further analyses.

† Logistic regression model was used to assess the association between 160-variant or Radial-filtered 126-variant fitness GRS and prevalent type 2 diabetes in the UK Biobank; the model was adjusted for age, sex, genotyping array, first 10 principal components; a total of 18,994 cases and 330,636 non-cases were included in the analyses.

**Supplementary Table 7. Mendelian Randomisation results for cardiorespiratory fitness on intermediate traits.**

| Outcome | Method | Radial-filtered | n_SNPs | betaIVW* | sebetaIVW | pIVW | CochQp |
|---|---|---|---|---|---|---|---|
| **Fasting Insulin** | Inverse variance weighted | No | 156 | -0.0073 | 0.0052 | 0.1602 | 0.00 |
| | MR Egger | No | 156 | -0.0135 | 0.0113 | 0.2363 | |
| | Egger Intercept | No | 156 | 0.0010 | 0.0017 | 0.5430 | |
| | Weighted median | No | 156 | -0.0129 | 0.0052 | 0.0144 | |
| | Inverse variance weighted | Yes | 134 | -0.0112 | 0.0032 | **0.0006** | 0.79 |
| | MR Egger | Yes | 134 | -0.0145 | 0.0068 | 0.0339 | |
| | Egger Intercept | Yes | 134 | 0.0006 | 0.0010 | 0.5624 | |
| | Weighted median | Yes | 134 | -0.0132 | 0.0051 | **0.0110** | |
| **Fasting glucose** | Inverse variance weighted | No | 156 | 0.0011 | 0.0057 | 0.8544 | 0.00 |
| | MR Egger | No | 156 | -0.0032 | 0.0125 | 0.7982 | |
| | Egger Intercept | No | 156 | 0.0007 | 0.0018 | 0.7019 | |
| | Weighted median | No | 156 | 0.0000 | 0.0043 | 1.0000 | |
| | Inverse variance weighted | Yes | 140 | -0.0002 | 0.0027 | 0.9260 | 0.98 |
| | MR Egger | Yes | 140 | -0.0038 | 0.0057 | 0.5059 | |
| | Egger Intercept | Yes | 140 | 0.0006 | 0.0008 | 0.4233 | |
| | Weighted median | Yes | 140 | 0.0000 | 0.0042 | 0.9980 | |
| **2-hour glucose** | Inverse variance weighted | No | 156 | -0.0157 | 0.0072 | 0.0293 | 0.00 |
| | MR Egger | No | 156 | 0.0042 | 0.0156 | 0.7877 | |
| | Egger Intercept | No | 156 | -0.0033 | 0.0023 | 0.1524 | |
| | Weighted median | No | 156 | -0.0120 | 0.0084 | 0.1537 | |
| | Inverse variance weighted | Yes | 141 | -0.0099 | 0.0051 | 0.0524 | 0.86 |
| | MR Egger | Yes | 141 | -0.0044 | 0.0122 | 0.7161 | |
| | Egger Intercept | Yes | 141 | -0.0009 | 0.0016 | 0.5947 | |
| | Weighted median | Yes | 141 | -0.0115 | 0.0078 | 0.1441 | |
| **HbA1c** | Inverse variance weighted | No | 155 | -0.0011 | 0.0048 | 0.8205 | 0.00 |
| | MR Egger | No | 155 | 0.0063 | 0.0104 | 0.5467 | |
| | Egger Intercept | No | 155 | -0.0012 | 0.0015 | 0.4257 | |
| | Weighted median | No | 155 | 0.0004 | 0.0049 | 0.9330 | |
| | Inverse variance weighted | Yes | 137 | -0.0009 | 0.0031 | 0.7753 | 0.74 |
| | MR Egger | Yes | 137 | 0.0046 | 0.0065 | 0.4840 | |
| | Egger Intercept | Yes | 137 | -0.0009 | 0.0009 | 0.3253 | |
| | Weighted median | Yes | 137 | 0.0005 | 0.0048 | 0.9248 | |
| **BMI** | Inverse variance weighted | No | 157 | -0.0033 | 0.0038 | 0.3883 | 0.00 |
| | MR Egger | No | 157 | -0.0012 | 0.0085 | 0.8925 | |
| | Egger Intercept | No | 157 | -0.0003 | 0.0012 | 0.7782 | |
| | Weighted median | No | 157 | -0.0002 | 0.0022 | 0.9411 | |
| | Inverse variance weighted | Yes | 108 | -0.0024 | 0.0015 | 0.1028 | 0.11 |
| | MR Egger | Yes | 108 | -0.0044 | 0.0031 | 0.1568 | |
| | Egger Intercept | Yes | 108 | 0.0003 | 0.0005 | 0.4698 | |
| | Weighted median | Yes | 108 | -0.0003 | 0.0022 | 0.8883 | |
| **SHBG** | Inverse variance weighted | No | 160 | 0.0092 | 0.0036 | 0.0106 | 0.00 |
| | MR Egger | No | 160 | -0.0012 | 0.0078 | 0.8772 | |
| | Egger Intercept | No | 160 | 0.0017 | 0.0011 | 0.1345 | |
| | Weighted median | No | 160 | 0.0027 | 0.0011 | 0.0173 | |
| | Inverse variance weighted | Yes | 99 | 0.0059 | 0.0008 | **9.6E-11** | 0.11 |
| | MR Egger | Yes | 99 | 0.0042 | 0.0019 | 0.0284 | |
| | Egger Intercept | Yes | 99 | 0.0003 | 0.0003 | 0.3090 | |
| | Weighted median | Yes | 99 | 0.0056 | 0.0013 | **2.4E-05** | |

*effect size are per unit higher of fitness (ml O2·$min^{-1}$·$kg^{-1}$ FFM).

**Supplementary Table 8. Multivariable Mendelian randomisation analysis results of cardiorespiratory fitness on type 2 diabetes adjusted for intermediate traits.**

| Covariate | n_snp | beta† | s.e. | p | p_heterogeneity |
|---|---|---|---|---|---|
| **None** | 126 | -0.017 | 0.006 | 0.005 | 0.686 |
| **Fasting Insulin (adj.BMI)** | 124 | -0.015 | 0.006 | 0.018 | 0.764 |
| **Fasting Glucose (adj.BMI)** | 124 | -0.018 | 0.006 | 0.003 | 0.907 |
| **2-hr Glucose (adj.BMI)** | 124 | -0.015 | 0.006 | 0.013 | 0.708 |
| **HbA1c** | 124 | -0.015 | 0.006 | 0.012 | 0.914 |
| **BMI** | 126 | -0.015 | 0.006 | 0.017 | 0.757 |
| **All 5 above intermediate traits** | 124 | -0.013 | 0.006 | 0.043 | 0.968 |
| **Sex hormone binding globulin** | 126 | -0.016 | 0.006 | 0.011 | |

*adj.BMI: adjusted for body mass index in the original genome-wide association analysis model for the intermediate trait

† beta values are log Odds of diabetes per ml O2·min$^{-1}$·kg$^{-1}$ FFM.

**Supplementary Table 9. Associations between the enhanced 160-SNP genetic risk score for cardiorespiratory fitness and Protein Targets assessed by the aptamer-based technology (SomaScan©) in 10,707 individuals from the Fenland study.**

| Target | TargetFullName | UniProtId | GeneName | beta | se | p |
|---|---|---|---|---|---|---|
| **N-terminal pro-BNP** | N-terminal pro B-type natriuretic peptide | P16860 | *NPPB* | 0.058 | 0.008 | 9.45E-13 |
| **MSP** | Hepatocyte growth factor-like protein | P26927 | *MST1* | -0.042 | 0.009 | 2.59E-06 |
| **MYL6B** | Myosin light chain 6B | P14649 | *MYL6B* | 0.036 | 0.009 | 4.66E-05 |
| **MXRA7** | Matrix-remodeling-associated protein 7 | P84157 | *MXRA7* | 0.033 | 0.009 | 1.26E-04 |
| **CD248** | Endosialin | Q9HCU0 | *CD248* | 0.033 | 0.009 | 1.93E-04 |
| **MYPC1** | Myosin-binding protein C, slow-type | Q00872 | *MYBPC1* | 0.032 | 0.009 | 2.29E-04 |
| **WISP-2** | WNT1-inducible-signaling pathway protein 2 | O76076 | *WISP2* | 0.031 | 0.009 | 4.24E-04 |
| **HS3S4** | Heparan sulfate glucosamine 3-O-sulfotransferase 4 | Q9Y661 | *HS3ST4* | 0.030 | 0.009 | 5.06E-04 |
| **MMP-2** | 72 kDa type IV collagenase | P08253 | *MMP2* | 0.031 | 0.009 | 5.21E-04 |
| **Apo B** | Apolipoprotein B | P04114 | *APOB* | 0.030 | 0.009 | 5.81E-04 |
| **MYOC** | Myocilin | Q99972 | *MYOC* | 0.028 | 0.008 | 6.33E-04 |
| **VPS29** | Vacuolar protein sorting-associated protein 29 | Q9UBQ0 | *VPS29* | -0.030 | 0.009 | 6.60E-04 |
| **SHBG** | Sex hormone-binding globulin | P04278 | *SHBG* | 0.026 | 0.008 | 9.86E-04 |
| **PAFAH** | Platelet-activating factor acetylhydrolase | Q13093 | *PLA2G7* | 0.029 | 0.009 | 9.91E-04 |

**Supplementary Table 10. DEPICT gene-set enrichment results for significant independent variants associated with cardiorespiratory fitness ($p<10^{-5}$).**

| Original gene set description | Nominal P value | FDR | First 10 genes in the reconstituted gene set (Z score) |
|---|---|---|---|
| **sequence-specific DNA binding RNA polymerase II transcription factor activity** | 2.25E-06 | **<0.05** | NR2F6 (3.0)\|C5orf41 (2.9)\|ACVR2B (2.5)\|ZFPM2 (2.4)\|LMX1B (2.4)\|ZNF219 (2.4)\|KIAA1267 (2.3)\|TNRC6B (2.2)\|WT1-AS (2.2)\|PVRL1 (2.2) |
| **RNA polymerase II core promoter proximal region sequence-specific DNA binding transcription factor activity involved in positive regulation of transcription** | 2.11E-05 | <0.20 | WT1-AS (3.8)\|ZFPM2 (2.9)\|LMX1B (2.6)\|ACVR2B (2.4)\|SGIP1 (2.2)\|MAPT (2.2)\|SMAD1 (2.1)\|BNC2 (2.1)\|ENSG00000224376 (2.0)\|THRB (1.7) |
| **muscle cell differentiation** | 2.84E-05 | <0.20 | C1orf105 (5.1)\|CCDC141 (3.9)\|SCN5A (3.8)\|SVIL (3.2)\|NKX2-5 (3.0)\|PLN (2.9)\|PLCE1 (2.9)\|MYH7 (2.6)\|TTN (2.6)\|KRT83 (2.5) |
| **muscle structure development** | 3.64E-05 | <0.20 | C1orf105 (6.3)\|CCDC141 (3.8)\|SCN5A (3.6)\|MYH7 (3.4)\|TTN (3.4)\|SVIL (2.9)\|NKX2-5 (2.9)\|PLN (2.8)\|MYH6 (2.6)\|PLCE1 (2.3) |
| **muscle tissue development** | 3.82E-05 | <0.20 | C1orf105 (5.8)\|SCN5A (4.4)\|CCDC141 (3.8)\|MYH7 (3.3)\|SVIL (3.2)\|NKX2-5 (3.1)\|MYH6 (2.9)\|TTN (2.9)\|PLCE1 (2.6)\|PLN (2.6) |
| **RNA polymerase II core promoter proximal region sequence-specific DNA binding transcription factor activity** | 4.58E-05 | <0.20 | WT1-AS (2.7)\|CDH11 (2.7)\|LMX1B (2.6)\|ACVR2B (2.3)\|SMAD1 (2.1)\|MAPT (2.0)\|TSLP (1.8)\|KIAA1267 (1.8)\|SOCS6 (1.8)\|DSP (1.8) |
| **RNA polymerase II distal enhancer sequence-specific DNA binding transcription factor activity** | 6.54E-05 | <0.20 | ENSG00000253744 (2.5)\|ZFPM2 (2.3)\|NKX2-5 (2.3)\|ARHGEF40 (2.2)\|KIAA1755 (2.1)\|HABP2 (2.0)\|DSP (1.9)\|BNC2 (1.9)\|PVRL1 (1.9)\|NR2F6 (1.9) |
| **striated muscle tissue development** | 7.50E-05 | <0.20 | C1orf105 (6.0)\|SCN5A (4.5)\|CCDC141 (4.1)\|NKX2-5 (3.4)\|MYH7 (3.4)\|MYH6 (3.0)\|TTN (3.0)\|SVIL (2.9)\|PLCE1 (2.7)\|OSBPL6 (2.6) |
| **negative regulation of cell migration** | 1.25E-04 | <0.20 | USHBP1 (2.8)\|ADAMTSL3 (2.7)\|ENSG00000224376 (2.0)\|CACNA1C (2.0)\|C3orf54 (1.9)\|MUC5B (1.9)\|PLEKHM1 (1.8)\|KIAA1755 (1.8)\|ADM (1.7)\|RASGRF2 (1.7) |
| **cell-matrix adhesion** | 1.26E-04 | <0.20 | CCDC141 (2.8)\|AMT (2.8)\|DAG1 (2.3)\|PLEKHA3 (2.3)\|FKBP7 (2.3)\|ADAMTSL3 (2.2)\|THRB-IT1 (2.2)\|USHBP1 (2.1)\|ENSG00000224376 (1.9)\|PLEC (1.9) |
| **striated muscle cell differentiation** | 1.34E-04 | <0.20 | C1orf105 (6.0)\|SCN5A (5.1)\|CCDC141 (4.5)\|NKX2-5 (3.7)\|PLN (3.5)\|KRT83 (3.3)\|SVIL (3.3)\|MYH7 (3.2)\|MYH6 (3.0)\|PLCE1 (2.8) |
| **muscle organ development** | 1.61E-04 | <0.20 | C1orf105 (6.9)\|SCN5A (3.7)\|CCDC141 (3.5)\|MYH7 (3.5)\|TTN (3.4)\|OSBPL6 (2.8)\|NKX2-5 (2.8)\|SVIL (2.8)\|MYH6 (2.5)\|PLN (2.3) |
| **increased cardiac muscle contractility** | 2.09E-04 | <0.20 | MYH7 (8.0)\|MYH6 (7.9)\|PLN (6.7)\|TTN (6.2)\|NKX2-5 (5.4)\|SCN5A (4.7)\|C1orf105 (4.1)\|CACNA1C (3.6)\|ENSG00000237975 (2.8)\|DSP (2.4) |

**Supplementary Table 11. MAGMA gene-set analysis using cardiorespiratory fitness GWAS summary statistics as implemented in FUMA.**

| Gene Set | N genes | Beta | Beta STD | SE | P | Pbon |
|---|---|---|---|---|---|---|
| **GO_bp:go_regulation_of_cardiac_muscle_contraction** | 70 | 0.65676 | 0.039576 | 0.11696 | 9.97E-09 | **1.54E-04** |
| **GO_bp:go_cardiac_muscle_contraction** | 125 | 0.40524 | 0.032585 | 0.08102 | 2.87E-07 | **4.45E-03** |
| **GO_bp:go_regulation_of_actin_filament_based_movement** | 40 | 0.68116 | 0.031053 | 0.14684 | 1.77E-06 | **2.74E-02** |
| **GO_bp:go_cardiac_muscle_cell_action_potential** | 63 | 0.51723 | 0.029574 | 0.11169 | 1.84E-06 | **2.84E-02** |
| **GO_bp:go_regulation_of_striated_muscle_contraction** | 84 | 0.46534 | 0.030706 | 0.10093 | 2.03E-06 | **3.14E-02** |
| GO_bp:go_striated_muscle_contraction | 158 | 0.31838 | 0.028757 | 0.071131 | 3.83E-06 | 5.93E-02 |
| GO_bp:go_regulation_of_heart_rate | 92 | 0.41899 | 0.028929 | 0.095165 | 5.38E-06 | 8.33E-02 |
| GO_bp:go_ventricular_cardiac_muscle_cell_action_potential | 32 | 0.72529 | 0.02958 | 0.16736 | 7.38E-06 | 1.14E-01 |
| GO_bp:go_cardiac_muscle_cell_contraction | 64 | 0.48413 | 0.0279 | 0.11321 | 9.55E-06 | 1.48E-01 |

* Bold gene sets were enriched based on Bonferroni corrected p-value

**Supplementary Table 12. Enriched gene-sets from various MSigDB resourses (FDR ≤ 0.05) using FUMA with 160 prioritised genes associated with cardiorespiratory fitness.**

| Database | GeneSet | N | n | P-value | adjusted P | Genes |
|---|---|---|---|---|---|---|
| **KEGG** | dilated cardiomyopathy | 89 | 6 | 7.1E-05 | 0.0133 | CACNA1C, MYH6, MYH7, TTN, ITGA4, PLN |
| **KEGG** | hypertrophic cardiomyopathy | 82 | 5 | 4.6E-04 | 0.0432 | CACNA1C, MYH6, MYH7, TTN, ITGA4 |
| **REACTOME** | muscle contraction | 206 | 11 | 6.9E-07 | 0.0010 | GUCY1A2, SLN, CACNA1C, MYH6, MYH11, TTN, SCN5A, SCN10A, SCN11A, NKX2-5, PLN |
| **GO BiologicalProcesses** | cardiac muscle contraction | 132 | 9 | 9.8E-07 | 0.0072 | CACNA1C, MYH6, MYH7, TTN, SCN5A, SCN10A, NKX2-5, PLN, GJA1 |
| **GO BiologicalProcesses** | striated muscle contraction | 166 | 9 | 6.5E-06 | 0.0239 | CACNA1C, MYH6, MYH7, TTN, SCN5A, SCN10A, NKX2-5, PLN, GJA1 |
| **GO BiologicalProcesses** | heart process | 278 | 11 | 1.2E-05 | 0.0305 | CACNA1C, MYH6, MYH7, TTN, SRC, THRB, SCN5A, SCN10A, NKX2-5, PLN, GJA1 |
| **Cancer gene modules** | module201 (B lymphoma) | 48 | 5 | 3.6E-05 | 0.0070 | MYH7, MYH11, SCN5A, PLN, GJA1 |
| **Cancer gene modules** | module387 (B lymphoma, breast cancer; liver cancer) | 49 | 5 | 4.0E-05 | 0.0070 | MYH7, MYH11, SCN5A, PLN, GJA1 |
| **Cancer gene modules** | module329 (B lymphoma; liver cancer) | 51 | 5 | 4.9E-05 | 0.0070 | MYH7, MYH11, SCN5A, PLN, GJA1 |

**Supplementary Table 13. DEPICT results for tissue and cell-type specific enrichment using significantly independent variants from cardiorespiratory fitness GWAS ($p < 10^{-5}$).**

| MeSH term | Name | MeSH 1st level term | MeSH 2nd level term | Nominal P value | FDR | Top 10 tissue-specific expressed genes (Z score) |
|---|---|---|---|---|---|---|
| **A07.541** | Heart | Cardiovascular System | Heart | 1.42E-04 | <0.05 | NR2F6 (3.0)\|C5orf41 (2.9)\|ACVR2B (2.5)\|ZFPM2 (2.4)\|LMX1B (2.4)\|ZNF219 (2.4)\|KIAA1267 (2.3)\|TNRC6B (2.2)\|WT1-AS (2.2)\|PVRL1 (2.2) |
| **A07.541.358** | Heart Atria | Cardiovascular System | Heart | 1.62E-04 | <0.05 | WT1-AS (3.8)\|ZFPM2 (2.9)\|LMX1B (2.6)\|ACVR2B (2.4)\|SGIP1 (2.2)\|MAPT (2.2)\|SMAD1 (2.1)\|BNC2 (2.1)\|ENSG00000224376 (2.0)\|THRB (1.7) |
| **A07.541.358.100** | Atrial Appendage | Cardiovascular System | Heart | 1.90E-04 | <0.05 | C1orf105 (5.1)\|CCDC141 (3.9)\|SCN5A (3.8)\|SVIL (3.2)\|NKX2-5 (3.0)\|PLN (2.9)\|PLCE1 (2.9)\|MYH7 (2.6)\|TTN (2.6)\|KRT83 (2.5) |
| **A07.541.560** | Heart Ventricles | Cardiovascular System | Heart | 4.65E-04 | <0.05 | C1orf105 (6.3)\|CCDC141 (3.8)\|SCN5A (3.6)\|MYH7 (3.4)\|TTN (3.4)\|SVIL (2.9)\|NKX2-5 (2.9)\|PLN (2.8)\|MYH6 (2.6)\|PLCE1 (2.3) |
| **A07.541.510.110** | Aortic Valve | Cardiovascular System | Heart | 3.53E-03 | <0.20 | C1orf105 (5.8)\|SCN5A (4.4)\|CCDC141 (3.8)\|MYH7 (3.3)\|SVIL (3.2)\|NKX2-5 (3.1)\|MYH6 (2.9)\|TTN (2.9)\|PLCE1 (2.6)\|PLN (2.6) |
| **A07.541.510** | Heart Valves | Cardiovascular System | Heart | 3.53E-03 | <0.20 | WT1-AS (2.7)\|CDH11 (2.7)\|LMX1B (2.6)\|ACVR2B (2.3)\|SMAD1 (2.1)\|MAPT (2.0)\|TSLP (1.8)\|KIAA1267 (1.8)\|SOCS6 (1.8)\|DSP (1.8) |
| **A05.360.319.679.690** | Myometrium | Urogenital System | Genitalia | 5.86E-03 | <0.20 | ENSG00000253744 (2.5)\|ZFPM2 (2.3)\|NKX2-5 (2.3)\|ARHGEF40 (2.2)\|KIAA1755 (2.1)\|HABP2 (2.0)\|DSP (1.9)\|BNC2 (1.9)\|PVRL1 (1.9)\|NR2F6 (1.9) |
