# Supplementary material for: Causal associations between cardiorespiratory fitness and type 2 diabetes
Source: Nat Commun. 2023 Jul 3;14:3904. doi: 10.1038/s41467-023-38234-w (PMC10318084; doi:10.1038/s41467-023-38234-w)
Supplement: Supplementary file 3 — Description of Additional Supplementary Files [file 41467_2023_38234_MOESM3_ESM.pdf]

Description of Additional Supplementary Files

File Name: Supplementary Data 1
Description: List of 160 variants included in the enhanced genetic risk score for fitness and effect sizes from genome-wide association analyses of fitness in UK Biobank (N=69,416).
