# Supplementary material for: Causal associations between cardiorespiratory fitness and type 2 diabetes
Source: Nat Commun. 2023 Jul 3;14:3904. doi: 10.1038/s41467-023-38234-w (PMC10318084; doi:10.1038/s41467-023-38234-w)
Supplement: Supplementary file 5 — Reporting Summary [file 41467_2023_38234_MOESM5_ESM.pdf]

nature research

Corresponding author(s): Nick Wareham, Soren Brage

Last updated by author(s): Mar 15, 2023

Reporting Summary

Nature Research wishes to improve the reproducibility of the work that we publish. This form provides structure for consistency and transparency in reporting. For further information on Nature Research policies, see our Editorial Policies and the Editorial Policy Checklist.

Statistics

For all statistical analyses, confirm that the following items are present in the figure legend, table legend, main text, or Methods section.

n/a	Confirmed	
☐	☒	The exact sample size (*n*) for each experimental group/condition, given as a discrete number and unit of measurement
☐	☒	A statement on whether measurements were taken from distinct samples or whether the same sample was measured repeatedly
☐	☒	The statistical test(s) used AND whether they are one- or two-sided<br>*Only common tests should be described solely by name; describe more complex techniques in the Methods section.*
☐	☒	A description of all covariates tested
☐	☒	A description of any assumptions or corrections, such as tests of normality and adjustment for multiple comparisons
☐	☒	A full description of the statistical parameters including central tendency (e.g. means) or other basic estimates (e.g. regression coefficient) AND variation (e.g. standard deviation) or associated estimates of uncertainty (e.g. confidence intervals)
☐	☒	For null hypothesis testing, the test statistic (e.g. *F*, *t*, *r*) with confidence intervals, effect sizes, degrees of freedom and *P* value noted<br>*Give P values as exact values whenever suitable.*
☒	☐	For Bayesian analysis, information on the choice of priors and Markov chain Monte Carlo settings
☒	☐	For hierarchical and complex designs, identification of the appropriate level for tests and full reporting of outcomes
☐	☒	Estimates of effect sizes (e.g. Cohen's *d*, Pearson's *r*), indicating how they were calculated

Our web collection on statistics for biologists contains articles on many of the points above.

Software and code

Policy information about availability of computer code

Data collection	All human population cohort data had been collected previously. Brief descriptions of the data collection for both UK Biobank and Fenland study cohorts can be found in the manuscript Methods section.
Data analysis	Main softwares used in the menuscript includes BOLT-LMM v2.3, LDSC, FUMA, DEPICT version 1 rel194, MetaXcan, STATA 15, MR-Base (http://www.mrbase.org), R packages 'TwoSampleMR' and 'MendelianRandomization'

For manuscripts utilizing custom algorithms or software that are central to the research but not yet described in published literature, software must be made available to editors and reviewers. We strongly encourage code deposition in a community repository (e.g. GitHub). See the Nature Research guidelines for submitting code & software for further information.

Data

Policy information about availability of data

All manuscripts must include a data availability statement. This statement should provide the following information, where applicable:

- Accession codes, unique identifiers, or web links for publicly available datasets
- A list of figures that have associated raw data
- A description of any restrictions on data availability

The genome-wide association study was conducted using UK Biobank resources. Fitness GWAS results in UK Biobank will be available through GWASCatalog at the time of publication. Other data supporting the findings can be found in the supplementary material.

Field-specific reporting

Please select the one below that is the best fit for your research. If you are not sure, read the appropriate sections before making your selection.

☒ Life sciences ☐ Behavioural & social sciences ☐ Ecological, evolutionary & environmental sciences

For a reference copy of the document with all sections, see nature.com/documents/nr-reporting-summary-flat.pdf

Life sciences study design

All studies must disclose on these points even when the disclosure is negative.

Sample size	Information about study sample size has been described in the Results and Methods section for observational and genomic analyses.
Data exclusions	Participants were excluded based on availability and quality of genotype and phenotype data. Details of exclusion criteria has been reported in the Methods section.
Replication	Various fitness-GRS constructed using 4 different criteria based on GWAS among UK Biobank participants were validated and prioritised in an independent cohort, the Fenland study, where all the fitness-GRS were significantly associated with measured fitness variable in the Fenland study.
Randomization	Randomization was not applicable in this study as there were no allocated experimental groups in quantitative genome-wide association studies among the population-based cohort.
Blinding	Blinding is not applicable in GWAS studies.

Reporting for specific materials, systems and methods

We require information from authors about some types of materials, experimental systems and methods used in many studies. Here, indicate whether each material, system or method listed is relevant to your study. If you are not sure if a list item applies to your research, read the appropriate section before selecting a response.

Materials & experimental systems

n/a	Involved in the study	
☒	☐	Antibodies
☒	☐	Eukaryotic cell lines
☒	☐	Palaeontology and archaeology
☒	☐	Animals and other organisms
☐	☒	Human research participants
☒	☐	Clinical data
☒	☐	Dual use research of concern

Methods

n/a	Involved in the study	
☒	☐	ChIP-seq
☒	☐	Flow cytometry
☒	☐	MRI-based neuroimaging

Human research participants

Policy information about studies involving human research participants

Population characteristics	The UK Biobank is a large-scale population-based cohort study including 503,325 participants (aged 40-69 years). The mean (± standard deviation) fitness level for the 34,179 men included in this analysis was 43.1 (± 6.4) ml O2·min-1·kg-1 FFM, and it was 39.8 (± 7.1) ml O2·min-1·kg-1 FFM for the 39,395 women (Supplementary Table 1). There were 1,852 cases of incident type 2 diabetes in the total sample of 73,574 people with fitness measurements during 10 years of follow-up. A total of 69,416 participants of European Ancestry who also have genetic data was included in the fitness GWAS and 452,941 participants were included in the resting heart rate GWAS in UK Biobank.<br><br>The Fenland Study is a population-based prospective cohort study that aims to investigate the associations between genetic and environmental factors and the risk of obesity, diabetes and related metabolic traits in adults. Exclusion criteria include clinically diagnosed diabetes mellitus, inability to walk unaided, terminal illness with prognosis ≤ 1 year at the time of recruitment, clinically diagnosed psychotic disorder, pregnancy or lactation. A total of 12,435 participants completed the baseline phenotype assessments including cardiorespiratory fitness, which was defined using estimated VO2max values derived from heart rate response during a submaximal treadmill test.
Recruitment	The UK Biobank participants were recruited through the general practitioners within the UK National Health Service between 2006 and 2016. Participants were enrolled in 22 study centres in England, Scotland and Wales, and provided extensive data on their demographic information, medical history and health behaviour through questionnaires.<br><br>Eligible participants for the Fenland study were born between 1950 and 1975, and resided in Cambridgeshire and were

registered at a participating General Practices in Cambridge, Ely, Wisbech and the surrounding Cambridgeshire region between 2004 and 2014.

Ethics oversight	The UK Biobank study was approved by the North West Multi-Centre Research Ethics Committee. All participants provided written informed consent.<br><br>The Fenland study was approved by the Cambridge Local Research Ethics Committee. All participants provided written informed consent.

Note that full information on the approval of the study protocol must also be provided in the manuscript.
